# A Right Turn in Diagnosis: Highlighting the Importance of TAPSE in Isolated Right Ventricular Myocardial Infarction

**DOI:** 10.24908/pocusj.v10i01.18433

**Published:** 2025-04-15

**Authors:** Vasudha Dinesh, Arun A. Mohanan, Amaravathi Uthayakumar, Vinodha Chandrashekar

**Affiliations:** 1 Department of Emergency Medicine, M. S. Ramaiah Medical College, Bangalore, IND; 2 Department of Emergency Medicine, JIPMER, Pondicherry, IND; 3 Department of Emergency Medicine, Sri Venkateshwaraa Medical College Hospital, Pondicherry, IND

**Keywords:** isolated right ventricular myocardial infarction, electrocardiography, tricuspid annular plane systolic excursion, point of care ultrasound, emergency medicine

## Abstract

Isolated right ventricular myocardial infarction (RVMI) is a rare but significant clinical entity that can present with atypical findings on a standard electrocardiograph (ECG). We present the case of a 65-year-old man with a history of chronic smoking and alcohol use who presented to the emergency department with acute chest pain. An initial ECG showed ST-segment elevation in lead V1 and depression in leads I, aVL, and V2-V6, which did not meet ST-segment elevation myocardial infarction (STEMI) criteria. A right-sided ECG revealed ST-segment elevation in V3R-V6R, concerning for RVMI. Notably, cardiac point of care ultrasound (POCUS) demonstrated normal left ventricular (LV) function without LV or right ventricular (RV) regional wall motion abnormalities (RWMA). However, tricuspid annular plane systolic excursion (TAPSE) was significantly reduced at 1 cm, indicating RV dysfunction despite the absence of RWMA. This is the first known case report that highlights the importance of TAPSE in assessing RV function in isolated RVMI cases. Early recognition and management are crucial, especially in patients with typical presentations, as prompt treatment can prevent complications. This case underscores the need for emergency physicians to maintain a high index of suspicion for RVMI, particularly in atypical presentations, and to utilize cardiac POCUS as an essential tool for evaluation.

## Introduction

Right ventricular myocardial infarction (RVMI) is a critical condition that can often go unnoticed, especially when patients do not present with classic symptoms or when conventional ST-segment elevation myocardial infarction (STEMI) criteria are not met. The importance of cardiac point of care ultrasound (POCUS) in the early identification of right ventricular (RV) dysfunction cannot be overstated, particularly in cases where left ventricular (LV) and RV regional wall motion abnormalities (RWMA) are absent. To our knowledge, this is the first case report that emphasizes the significance of measuring tricuspid annular plane systolic excursion (TAPSE) by cardiac POCUS to assess RV function in a case of isolated RVMI.

## Case Presentation

A 65-year-old man with a history of chronic tobacco and alcohol use but no known comorbidities presented to the emergency department with acute chest pain lasting three hours. The pain was central, tight, and radiated to the left arm, and was accompanied by profuse sweating.

**Figure 1.**
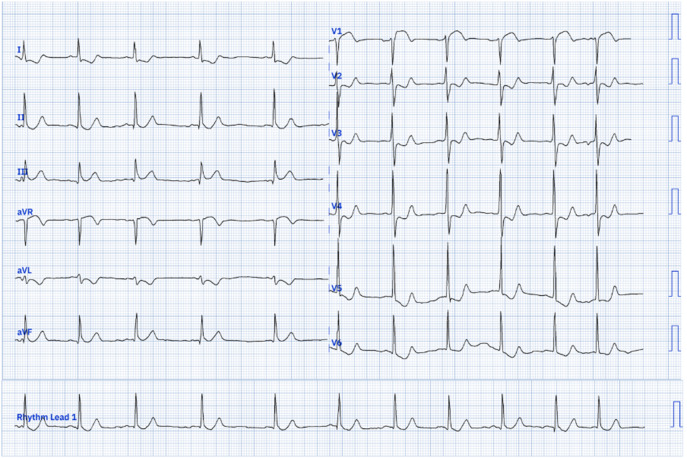
ECG showing ST elevation in lead V1 and ST depression in leads 1,avL,V2-V6

**Figure 2.**
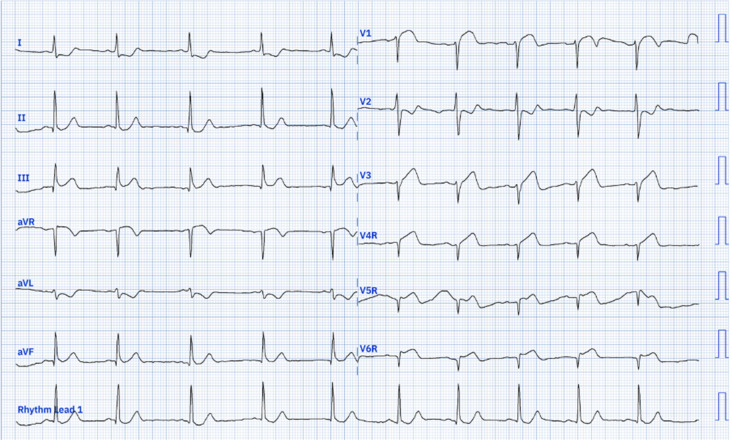
Right sided ECG showing ST elevations in V4R-V6R

**Figure 3.**
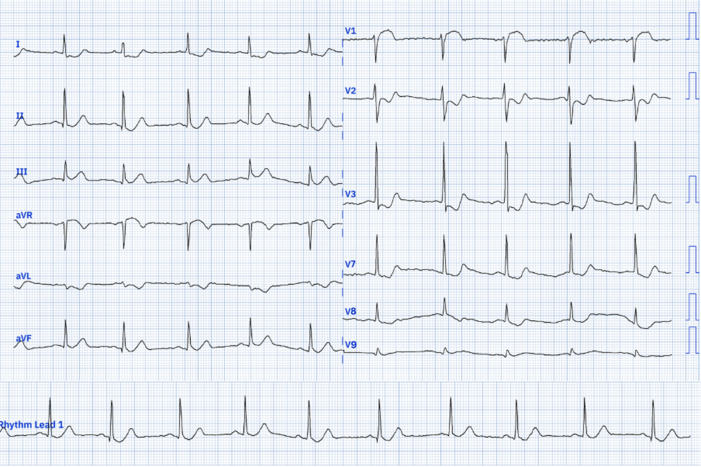
Posterior ECG showing no elevations in V7-V9

**Figure 4.**
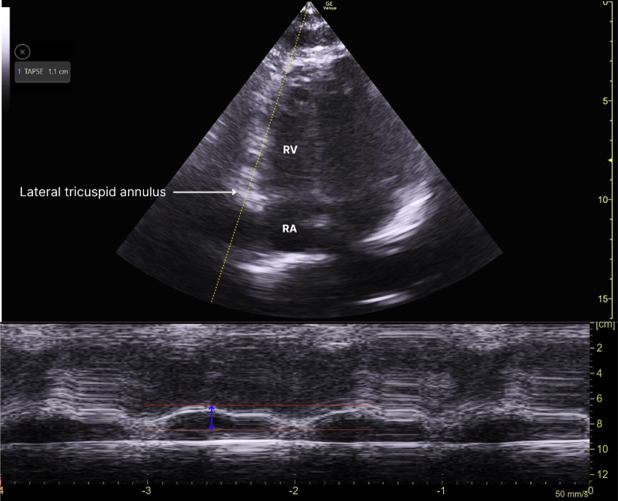
POCUS image showing TAPSE of 1.1cm

Upon examination, his pulse rate was 78 bpm, and his blood pressure was 120/70 mmHg in the right upper limb and 110/70 mmHg in the left. The systemic examination was unremarkable. A 12-lead electrocardiograph (ECG) revealed a heart rate of 76/min with normal sinus rhythm, ST-segment elevation in V1, and ST-segment depression in leads I, aVL, V2 to V6 (Figure 1). According to the fourth universal definition of myocardial infarction (2018), the ECG did not meet the STEMI criteria since ST-segment elevation was not present in two contiguous leads [1]. Due to the typical presentation of chest pain and ST-segment elevation in lead V1, a right-sided and posterior ECG was obtained (Figures 2 and 3), confirming ST-segment elevation from V3R to V6R, indicative of RVMI. Cardiac POCUS was performed, revealing a normal LV ejection fraction with no LV or RV RWMA. However, with a high degree of suspicion for RVMI, TAPSE was measured, yielding a value of 1.1 cm (Figure 4) (normal > 1.7 cm), suggesting RV dysfunction [2].

The patient received analgesics, Aspirin 325 mg, Clopidogrel 300 mg, and Atorvastatin 80 mg.

Since primary percutaneous coronary intervention (PCI) was not feasible, the patient was thrombolysed. PCI performed one week later revealed a 70% occlusion of the right coronary artery.

## Discussion

Isolated RVMI is a rare and often underdiagnosed condition, comprising a small percentage (<3%) of myocardial infarction [3,4]. Only 19 cases have been reported as of 2022 [3]. Unlike typical myocardial infarctions that primarily affect the left ventricle, RVMI can present with subtle signs and symptoms that may be easily overlooked, especially in the absence of LV or RV RWMA on initial imaging. Identifying RV dysfunction is critical because it has significant implications for patient management and treatment strategies [5]. Emergency physicians play a crucial role in recognizing this condition, as prompt identification can significantly influence management strategies and patient outcomes. Early recognition of RVMI enables targeted therapies, such as thrombolysis or PCI, which can reduce mortality.

The use of right-sided ECG leads is crucial in diagnosing RVMI, as it can reveal ST-segment changes that are not evident on standard leads [6,7,8]. Cardiac POCUS provides immediate assessment of cardiac function, allowing clinicians to evaluate TAPSE and assess RV dysfunction effectively. In RVMI, the absence of RV or LV RWMA on conventional imaging does not rule out the presence of myocardial injury. This is particularly relevant in cases where RV function may be compromised despite normal LV wall motion. TAPSE measurement via POCUS provides essential insight into RV performance. A decreased TAPSE value, as seen in this case, is indicative of RV dysfunction. This may occur even when the left ventricle appears to be functioning normally.

## Conclusion

In recent years, awareness of RVMI and the utilization of TAPSE through cardiac POCUS have gained significance in emergency medicine. Understanding the limitations of traditional imaging and the capabilities of cardiac POCUS will enable better identification of myocardial infarction and facilitate timely intervention. Emergency physicians should be proficient in these diagnostic modalities to improve outcomes in patients presenting with acute chest pain.



## References

[1] Thygesen K, Alpert JS, Jaffe AS, Chaitman BR, Bax JJ, Morrow DA, White HD; Executive Group on behalf of the Joint European Society of Cardiology (ESC)/American College of Cardiology (ACC)/American Heart Association (AHA)/World Heart Federation (WHF) Task Force for the Universal Definition of Myocardial Infarction. Fourth Universal Definition of Myocardial Infarction (2018). J Am Coll Cardiol. 2018 Oct 30;72(18):2231–2264. doi: 10.1016/j.jacc.2018.08.1038. Epub 2018 Aug 25. PMID: 30153967. 30153967

[2] Modin D, Mogelvang R, Andersen DM, Biering-Sorensen T. Right Ventricular Function Evaluated by Tricuspid Annular Plane Systolic Excursion Predicts Cardiovascular Death in the General Population. J Am Heart Assoc 2019;8:e012197. 31088196 10.1161/JAHA.119.012197 PMC6585329

[3] Hiraya D, Sato A, Watabe H, Hoshi T, Ieda M. Isolated Right Ventricular Infarction: A Case Report and Literature Review. Intern Med 2022;61:495–500. 34433722 10.2169/internalmedicine.7920-21 PMC8907765

[4] Finn AV, Antman EM. Isolated Right Ventricular Infarction. New England Journal of Medicine 2003;349:1636–1636. 14573735 10.1056/NEJMicm030315

[5] Baydar O, Oktay V, Coskun U, Yildiz A, Gurmen T. Isolated Right Ventricular Infarction Mimicking Anterior ST-Segment Elevation. J Clin Diagn Res. 2016 Apr;10(4):OD08–9. doi: 10.7860/JCDR/2016/17490.7599. Epub 2016 Apr 1. PMID: 27190867; PMCID: . 27190867 PMC4866165

[6] Harnett DT, LaHaye SA, Wilkinson JS. Isolated Right Ventricular Myocardial Infarction: A Sheep in Wolf's Clothing. JAMA Intern Med. 2016 Aug 1;176(8):1207–10. doi: 10.1001/jamainternmed.2016.3085. PMID: 27367171. 27367171

[7] Marques A, Cruz I, Briosa A, João I, Almeida S, Pereira H. Isolated Right Ventricle Myocardial Infarction - Is the Right Ventricle Still the Forgotten Ventricle? Arq Bras Cardiol. 2021 Feb;116(2 suppl 1):32–35. English, Portuguese. doi: 10.36660/abc.20200164. PMID: 33567001; PMCID: . 33567001 PMC8118633

[8] Arai R, Murata N, Yamada A, Monden M, Morikawa T, Akutsu N, Fukamachi D, Okumura Y. Pitfall of Isolated Right Ventricular Infarction Caused by Non-Dominant Right Coronary Artery. Circ J. 2021 May 25;85(6):956. doi: 10.1253/circj.CJ-21-0108. Epub 2021 Apr 7. PMID: 33828022. 33828022

